# Instruments Measuring Blunted Affect in Schizophrenia: A Systematic Review

**DOI:** 10.1371/journal.pone.0127740

**Published:** 2015-06-02

**Authors:** Sanja Kilian, Laila Asmal, Anneke Goosen, Bonginkosi Chiliza, Lebogang Phahladira, Robin Emsley

**Affiliations:** Department of Psychiatry, Faculty of Medicine and Health Sciences, Stellenbosch University, Tygerberg, South Africa; Maastricht University Medical Centre, NETHERLANDS

## Abstract

Blunted affect, also referred to as emotional blunting, is a prominent symptom of schizophrenia. Patients with blunted affect have difficulty in expressing their emotions. The work of Abrams and Taylor and their development of the Rating Scale for Emotional Blunting in the late 1970’s was an early indicator that blunted affect could indeed be assessed reliably. Since then, several new instruments assessing negative symptoms with subscales measuring blunted affect have been developed. In light of this, we aim to provide researchers and clinicians with a systematic review of the different instruments used to assess blunted affect by providing a comparison of the type, characteristics, administration and psychometric properties of these instruments. Studies reporting on the psychometric properties of instruments assessing blunted affect in patients with schizophrenia were included. Reviews and case studies were excluded. We reviewed 30 full-text articles and included 15 articles and 10 instruments in this systematic review. On average the instruments take 15–30 minutes to administer. We found that blunted affect items common across all instruments assess: gestures, facial expressions and vocal expressions. The CAINS Self-report Expression Subscale, had a low internal consistency score. This suggests that this sub-scale does not reliably assess patients’ self-reported blunted affect symptoms and is likely due to the nature of blunted affect. Instruments correlated minimally with instruments measuring positive symptoms and more importantly with depression suggesting that the instruments distinguish between seemingly similar symptoms.

## Introduction

Blunted affect, also referred to as emotional blunting, is a prominent symptom of schizophrenia. Patients with blunted affect have difficulty in expressing their emotions [1], characterized by diminished facial expression, expressive gestures and vocal expressions in reaction to emotion provoking stimuli [1–3]. However, patients’ reduced outward emotional expression may not mirror subjective internal emotional experiences [4] suggesting a disconnect in what patients experience, perceive and express when interpreting emotional stimuli [5] due to problems associated with emotional processing [6–7].

Blunted affect is regarded as a negative symptom of schizophrenia and like other negative symptoms, our assessment, treatment and overall understanding of blunted affect remains limited [8–9]. There are many factors that should be considered when assessing blunted affect. For example, blunted affect can co-vary with the negative symptom alogia [10], but whether these symptoms involve the same or a different neurobiological basis remains unanswered. Blunted affect can be a feature of both primary and secondary negative symptoms [1]. In schizophrenia, blunted affect closely resembles symptoms of depression making it difficult to distinguish between the two in the absence of appropriate instruments [11]. Symptoms such as lack of facial expression, apathy and psychomotor slowing are often associated with both. Also, antipsychotic medications as well as antidepressants can cause secondary blunted affect [1, 10]. It is therefore suggested by Kirkpatrick [1] that the Schedule for Deficit Syndrome (SDS) be considered in conjunction with negative symptoms instruments in order to assist in determining whether blunted affect symptoms are primary or secondary. The reason being that patients who are diagnosed with deficit syndrome are more likely to experience primary enduring blunted affect symptoms [1].

While blunted affect is an integral component of the symptom expression of the illness it is not unique to schizophrenia. Indeed, in degenerative disorders such as Parkinson’s disease blunted affect is frequently a prominent symptom. Also, antipsychotic-induced parkinsonism, which has a similar clinical presentation as Parkinson’s disease, may cause blunted affect which is clinically indistinguishable from blunted affect due to schizophrenia. In all of these disorders the fronto-striatal reward pathways are thought to play a role in producing blunted affect [12–13].

The abovementioned factors may complicate the assessment of blunted affect. The work of Abrams and Taylor and their development of the Scale for Emotional Blunting (SEB) in the late 1970’s was an early indicator that blunted affect could be assessed reliably [14]. Since then, several new instruments assessing negative symptoms with subscales measuring blunted affect have been developed. In light of this, this paper focusses on blunted affect and the clinical assessment thereof. We aim to provide researchers and clinicians with a systematic review of the different instruments used to assess blunted affect by providing a comparison of the type, characteristics, administration and psychometric properties of these instruments.

## Methods

### Study selection and data extraction

#### Inclusion criteria

- Studies reporting on the psychometric properties of uni- or multidimensional instruments assessing blunted affect in patients with schizophrenia.
- Description of the psychometric properties of the instrument in a peer-reviewed journal.
- Studies published in English.
- A study population of 16 years and older.

#### Exclusion criteria

- Reviews and case studies were excluded.

#### Search strategy

The first author, SK, performed the search between July to October 2013 and searched PubMed and PsycArticles databases. SK selected additional studies by cross-checking article references. The following search terms were used: ‘*blunted affect*’ or ‘*emotional blunting*’ AND ‘*schizophrenia*’ AND ‘*scale*’ OR ‘*rating scale*’ OR ‘*interview*’ OR ‘*questionnaire*’ OR ‘*instrument*’ AND ‘*reliability*’ OR ‘*validation*’. The PubMed search was performed using standardised medical subject search terms (Mesh) and all logical subsets (synonyms) were also included in the search. No filters were included.

SK compiled a list of possible studies to include in this review based on the abstracts that appeared to meet the inclusion criteria of the review. SK and AG individually reviewed the list of possible studies and assessed the full text of relevant articles for eligibility. In addition, two raters independently assessed the quality of the full-text articles using the *Quality Assessment of Diagnostic Accuracy Studies* 2 (QUADAS-2) tool [15]. Together SK and AG decided on the final list of studies included in the review. Study authors were contacted for further information where necessary. Information was extracted on study characteristics, populations, psychometric properties and the general characteristics of the various instruments reported in the full-text articles.

### Psychometric properties: reliability and validity

A reference (gold) standard provides the classification to determine whether a target condition is present in diagnostic accuracy studies. A key challenge in many psychiatric conditions, including in the assessment of blunted affect in schizophrenia is that there is no gold standard instrument. We therefore evaluated the psychometric properties of each index instrument (extracted by SK) by systematically evaluating their reliability and validity.

#### Reliability

Reliability was evaluated by referring to the reported test-retest reliability, internal consistency, and inter-scorer reliability. Test-retest reliability refers to the correlation between the test and retest results. Internal consistency (also known as inter-item consistency) refers to the consistency with which responses are provided to every item of the instrument. Inter-scorer reliability refers to the correlation between the scoring of the instrument by different instrument administraters [16]. In this systematic review we regard a value of between 0.60–0.70 as acceptable, and above 0.70–0.90 as good for reliability and intra-class coefficient (ICC) measures. [17].

#### Validity

We evaluated the validity of the instruments by referring to the reported convergent (also referred to as concurrent validity), divergent (also known as discriminant validity), and predictive validity of the instruments. An instrument has convergent validity when the information it captures correlates with the information captured by another instrument measuring the same construct [18]. An instrument has divergent validity when it correlates minimally with instruments which measure other symptoms [16–17].

## Results

### Studies included

**Fig 1:**
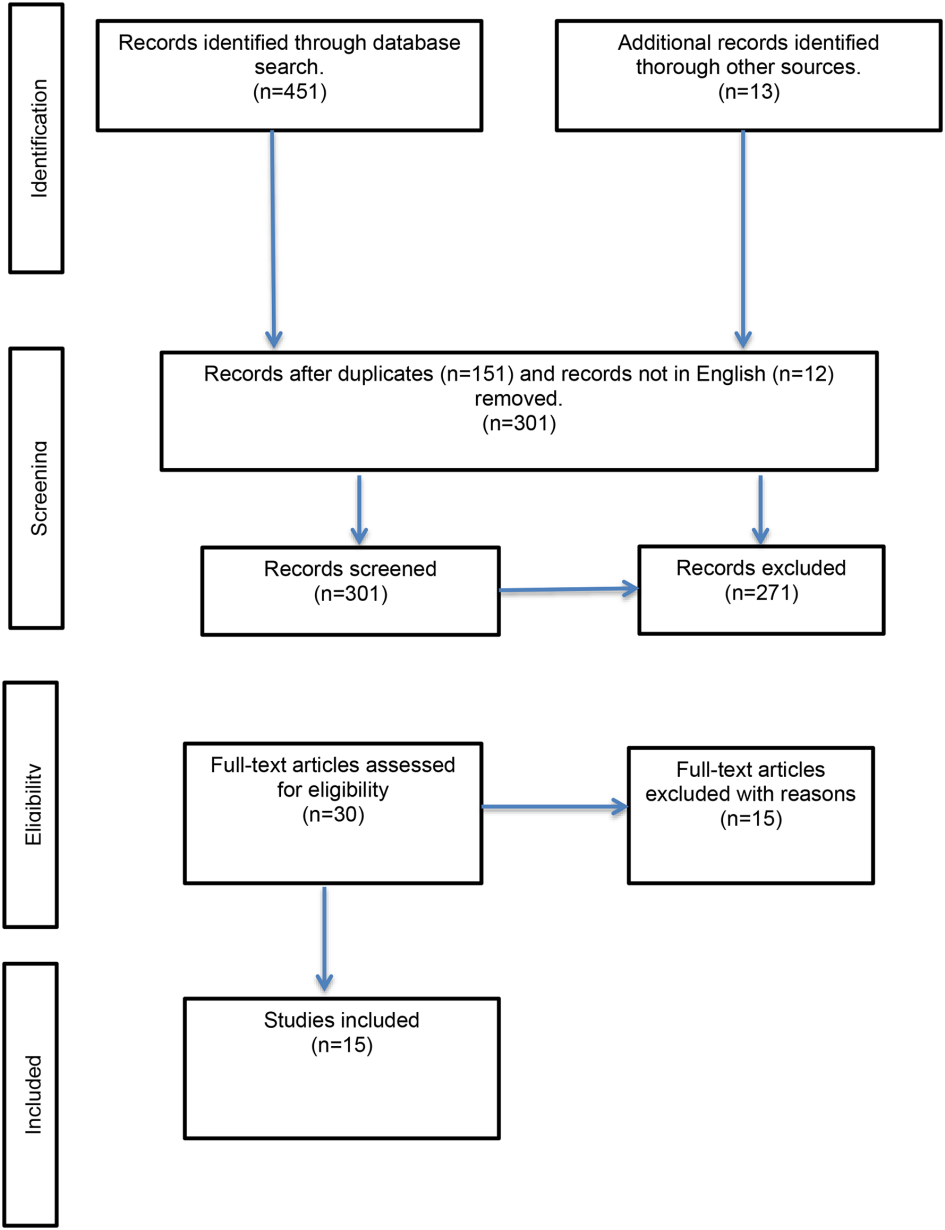
PRISMA Flow diagram. Flow diagram of selected studies.

Fig 1 illustrates the selection process we underwent to select the studies to be included for this paper. We report on the selected items according to the PRISMA (Preferred Reporting Items for Systematic Reviews and Meta-Analyses) guidelines (S1 PRISMA Checklist).

We found 451 records through the databases search and 13 additional records thorough cross-referencing checks. We removed 151 records due to duplication and 12 records that were not available in English. Of the remaining 301 records, we excluded 271 records that did not report on the psychometric properties of measurement instruments. We reviewed 30 full-text articles and excluded 15 due to the following reasons: studies did not investigate the instrument’s divergent or convergent validity (n = 13); and articles reported on instruments in their developmental phase (n = 2). One of the instruments, the Negative Symptom Assessment Short (NSA-4), was excluded since it did not include items assessing blunted affect. In the end we included 10 negative symptom instruments with items measuring blunted affect. The 10 instruments are:

1. Brief Negative Symptoms Scale (BNSS) [19–20];
2. Brief Psychiatric Rating Scale (BPRS) [21];
3. Clinical Assessment Interview for Negative Symptoms (CAINS) [22];
4. Clinical Assessment Interview for Negative Symptoms Self-report (CAINS-SR) [23];
5. Negative Symptom Assessment (NSA-16) [24–25];
6. the Negative Symptom Subscale of the Positive and Negative Syndrome Scale (PANSS-N) [26–27];
7. the Negative Symptom Subscale of the Positive and Negative Symptom Questionnaire (PNS-Q) [28];
8. Scale for the Assessment of Negative Symptoms (SANS) [29–30];
9. Motor Affective Social Scale (MASS) [31]; and
10. Scale for the Assessment of Emotional Blunting (SEB) [32–34].

### Instrument type, characteristics and administration

All instruments are clinician-rated, except for two self-report instruments (CAINS-SR and PNS-Q). Also, the MASS has two sections. In one part of the MASS the clinician count specific patient behaviours during a structured interview and in the second part ward staff are interviewed and asked to report on the patient’s *grooming and hygiene*, *participation in group activities*, *verbal interaction with staff*, and *spontaneous motor activity*. On average the instruments take 15–30 minutes to administer.

All the instruments are multi-dimensional measures of negative symptoms (for a complete list of the blunted affect items included in each instrument (S1 Table). The name of the SEB seems to suggest that it is a unidimensional instrument. However, as noted by De Leon et al. [33] the concept ‘emotional blunting’ as rated by the SEB also includes items measuring avolition and anhedonia.

Some of the subscales measuring blunted affect also include the item *reduced quantity of speech* which measures alogia rather than blunted affect. This is in line with more recent developments (i.e. the NIMH Consensus Development Conference) measuring blunted affect and alogia as to some extent independent symptoms. The majority of instruments (BNSS, CAINS, CAINS-SR, PANSS-N, NSA-16) measure blunted affect exclusively through three items: patients’ facial and vocal expressions of emotion as well as their expressive gestures. The BPRS has three items measuring similar aspects: *reduced emotional tone; reduction in formal intensity of feelings*; and *flatness*.

In contrast the SANS, PNS-Q and SEB include more than three blunted affect items. In addition to the three items assessing *facial and vocal expression* and *expressive gestures*, the SANS and PNS-Q also include the items of *poor eye contact* and *inappropriate affect*. Rabany et al. [29] performed a factor analysis of the structure of the SANS and found that poor eye contact did not load on the blunted affect factor (or any negative factor) and suggests that this item should be dropped from the blunted affect subscale. Also, De Leon (34) found that *inappropriate affect* should not be included as an item measuring blunted affect. The original SEB [14] consists of 16 items described as items measuring emotional blunting. However, as noted by De Leon et al. [33] the concept ‘emotional blunting’ as rated by the SEB also includes items measuring avolition and anhedonia. Two studies explored the factor structure of the SEB and derived somewhat different blunted affect subscales. Berenbaum et al. [34] suggest that the SEB blunted affect subscale consists of the following items:

*absent*, *shallow*, *incongruous mood*;

*constricted affect*;

*unvarying affect*;

*expressionless face*;

*unvarying monotonous voice*; and

*difficulty to excite emotions*.

In comparison De Leon et al. [33] include five of the items in Berenbaum’s blunted affect subscale (but exclude the item *absent*, *shallow*, *incongruous mood)* and in addition the items *spontaneity*, *paucity of thought* and *unrelated affect* are included. The MASS takes a slightly different approach to assessing blunted affect insofar as it counts displayed co-verbal gestures and spontaneous smiles during the clinical interview. In addition patients’ posed smiles are assessed at the end of the clinical interview.

### Psychometric properties

In this section we contextualize the study findings by first reporting on the psychometric properties of the overall negative symptom instruments. Thereafter we describe the pyschometric properties of blunted affect subscales with sufficient reported information.

#### Reliability and Validity of Overall Negative Symptoms Instruments

Table 1 details the reliability of the overall negative symptom instruments based on the instruments’ total score. Instruments with reported internal consistency (BNSS, CAINS, NSA-16, MASS, PANSS-N, PNS-Q-N, SANS, SEB) had good to excellent internal consistency values, ranging 76–94. We found that there was a noteworthy difference between the internal consistency of the BNSS (with a value ranging between 0.93–0.94) while the CAINS had a value of 0.76. The difference in internal consistency cannot be ascribed to scale length as both instruments have 13 items. Also the instruments were tested in samples with similar characteristics. The BNSS [20] was tested in 100 clinically stable patients with schizophrenia (n = 88) and schizoaffective disorder (n = 12) with a mean age of 42.2 years (SD = 11.1), mainly treated with second generation antipsychotics (i.e. 79%). The CAINS [22] was tested in 162 moderately ill outpatients with schizophrenia (n = 139) and schizoaffective disorder (n = 23) with a mean age of 36.8 years (SD = 9.5), mainly treated with second generation antipsychotics (i.e. 71%). This said, the developers, Kring et al., of the CAINS found that when the subscales were tested they had a higher internal consistency than the overall instrument [22].

**Table 1:** Reliability of negative symptom instrument.

Instrument	Source	Scale Items	Sample	Internal Consistency	Inter-rater Reliability	Test-retest Interval	Test-retest Reliability
**BNSS**	[19]	13	20	0.93, p<0.001	0.96	1 week	0.81, p<0.001
	[20]		100	0.94, p<0.001^1^	-	56–371 days	0.93, p<0.001
**BPRS**	[21]	18	47	-	0.90	-	-
**CAINS**	[22]	13	162	0.76	-	1 day-6months	0.69
**CAINS SR**	[23]	30	69	0.51, p<0.01	-	-	-
**NSA-16**	[24]	16	223	0.92	-	-	-
	[25]		561	0.85	-	-	0.87
**MASS**	[31]	8	101	0.81	-	1–2 weeks	0.85
**PANSS-N**	[27]	7	100	0.92, p<0.001	0.80^2^	-	-
	[26]		101	0.83, p<0.001	-	3–6months	0.68, p<0.01^3^
**PNS-Q Negative Subscale**	[28]	68	61	0.89	-	-	-
**SANS**	[29]	25	240	0.88	-	-	-
	[30]		207	0.89	-	-	-
**SEB**	[32]	16	40	-	0.90, p<0.01	-	0.87, p<0.01^4^
	[33]		115	0.90	0.44–0.95^5^	-	-

Reliability estimates of negative symptom instruments based on total scores.

^1^All scale items, except for one anhedonia item, were statistically significant

^2^Inter-rater reliability tested in subsample (n = 27)

^3^Test-retest reliability tested in subsample (n = 15)

^4^Test-retest reliability tested in subsample (n = 20)

^5^13 of 16 items had ICC of 0.75 or higher

Instruments with reported interrater-reliability (BNSS, BPRS, PANSS-N, SEB) had good to excellent values ranging between 80–96. All instruments with reported test-retest reliability (BNSS, NSA-16, MASS, PANSS-N, SEB), except for the PANSS-N, had good to excellent test-retest reliability (values ranging from 81–93). The PANSS-N had a test-retest reliability value of 0.68, which is still considered acceptable. Therefore none of the instruments, based on the instruments’ overall values, had poor reliability estimates.

In Table 2 we provide information pertaining to overall negative symptom instruments validity. Instruments with reported convergent validity (i.e. BNSS, NSA-16, MASS, PANSS-N, PNS-Q-N, SANS, and SEB) had values ranging from moderate to excellent, indicating that they correspond well with other negative symptom instruments. One study reported on the use of the Schedule for Deficit Syndrome (SDS) in conjunction with negative symptom instruments. The study conducted by Strauss et al. [20] found that schizophrenia patients with deficit disorder (as rated by the SDS) had higher BNSS scores than non-deficit patients [20].

**Table 2:** Validity of overall negative symptom instrument.

Instrument	Source	Convergent Validity	Divergent Validity	Predictive Validity
**BNSS**	[19]	0.84 p< 0.001 (SANS); 0.80, p< 0.001 (PANSS-N)	0.09 (PANSS-P); 0.14 (PANSS-D)	0.60, p<0.001 (CGI)
	[20]	0.80, p<0.001 (SANS); 0.68, p<0.001 (BPRS-N)	-0.06 (BPRS-P); 0.04 (BPRS-Disorganization); 0.32, p<0.01 (BPRS Total)	
**NSA-16**	[25]	0.59 (0.51, 0.66) (PANSS-N)	0.23 (0.13,0.33) (PANSS P Marder factor); -0.10 (CDSS)	0.91 (0.89, 0.93) (CGI-S); 0.83 (0.81, 0.86) (NSA-Global)
**MASS**	[31]	-0.79, p<0.0001 (SANS); -0.80, p<0.0001 (PANSS-N)	0.22, p = 0.03 (PANSS-P); -0.05, p = 0.61 (MADRS)	-
**PANSS-N**	[27]	-0.81 (SANS)	-	-
**PNS-Q (Negative Symptom Subscale)**	[28]	-0.086, p>0.1 (SANS)	0.206 (SAPS)	-
**SANS**	[29]	0.56 (PANSS-N)	-0.18 (CDSS)	-
**SEB**	[32]	0.75 (SANS-EB)	-	-
	[33]	0.76(SANS-EB)	-	-

Validty estimates of negative symptom instruments based on total scores.

BPRS-N (Brief Psychiatric Rating Scale Negative Symptoms Subscale); BPRS-P (Brief Psychiatric Rating Scale Positive Symptoms Subscale); BPRS-D (Brief Psychiatric Rating Scale Disorganization Subscale); CDSS (Calgary Depression Scale Schizophrenia); CGI-S (Clinical Global Impression Severity Scale); PANSS-P (Positive Negative Symptom Scale Positive Symptoms Subscale); PANSS-D (Positive Negative Symptom Scale Depressive Symptoms Subscale); SANS-EB (Scale for the Assessment of Negative Symptoms Blunted affect Subscale).

In terms of divergent validity there was a minimal correlation, due to low scores, between instruments and the PANSS Positive Symptom Marder Factor and the CDSS. This suggests that the instruments do not measure positive symptoms or depression. The BNSS and the NSA-16 were the only instruments with reported information pertaining to predictive validity. The BNSS had acceptable and the NSA-16 had good predictive validity.

#### Reliability and Validity of Blunted Affect Subscales

Detail pertaining to the psychometric properties of the blunted affect subscales is presented in Tables 3–5. Studies described the reliability of blunted affect subscales of the following negative symptom instruments: BNSS, CAINS, CAINS-SR, PANSS-N, PNS-Q-N and SANS (see Table 3). The blunted affect subscales of the CAINS and SANS have good (0.88) internal consistency estimates. While the blunted affect subscales of the PANSS-N’s and PNS-Q-N have acceptable (respectively 0.63 and 0.60) internal consistency, and the CAINS-SR Expression has poor (0.44) internal consistency. The developers of the CAINS-SR explain that the poor internal consistency may be due to patients’ limited awareness of their expression of emotion [23]. The BNSS Blunted Affect Subscale and CAINS Expression have respectively excellent (0.92) and good (0.77) inter-rater reliability. The test-retest reliability of the BNSS blunted affect subscale was good (0.77) and that of the CAINS Expression was acceptable (0.69). Similarly to the reliability of the overall negative symptom instruments, the subscales have acceptable to excellent reliability scores. The only subscale with less than optimal reliability is the CAINS-SR Expression Subscale that we referred to above.

**Table 3:** Reliability of subscales measuring blunted affect.

Instrument	Source	Sub-scale items	Internal Consistency	Inter-rater Reliability	Test-retest Reliability
**BNSS Blunted Affect Subscale**	[19]	3	-	0.92	0.77, p<0.001
	[20]	3	-0.84–0.87, p<0.001	-	-
**CAINS Expression Subscale**	[22]	3	0.88	0.77	0.69
**CAINS Self-Report Expression Subscale**	[23]	5	0.44	-	-
**PANSS-N Blunted Affect Subscale**	[26]	1^6^	0.63, p<0.001	-	-
	[27]	1	0.85, p<0.001	0.75, p<0.001	-
**PNS-Q Blunted Affect Subscale**	[28]	7	0.60	-	-
**SANS Blunted Affect Subscale**	[29]	8	0.88	-	-
	[30]	8	0.93	-	0.54, p<0.001
**SEB Blunted Affect Subscale**	[33]	8	0.84	-	-
	[34]	6	0.86	-	-

Reliability estimates of negative symptom instruments’ subscales measuring blunted affect.

^6^ The emotional blunting item refers to diminished emotional responsiveness as observed through a reduction in facial expression, modulation of feelings, and communicative gestures. Observations are made of the physical manifestations of affective tone and emotional responsiveness during the course of interview

**Table 4:** Convergent validity of subscales measuring blunted affect.

Instrument	Source	SANS Blunted Affect	BPRS-N	SANS Total	CAINS-Expression Subscale	PANSS-N
**BNSS Blunted Affect Subscale**	[19]	0.79, p<0.001	-	-	-	-
**BPRS Anergia Subscale**	[21]	0.66, p<0.001	-	-	-	-
**CAINS Expression Subscale**	[22]	0.61, p<0.01	0.52, p<0.01	0.55, p<0.05	-	-
**CAINS-SR Expression Subscale**	[23]	-	-	-	0.29, p<0.05	-
**SANS Blunted Affect Subscale**	[29]	-	-	-	-	0.45, p<0.01
**SEB Blunted Affect Subscale**	[33]	0.76	-	-	-	-

Convergent validity estimates of negative symptom instruments’ subscales measuring blunted affect.

**Table 5:** Divergent validity of subscales measuring blunted affect.

Instrument	Source	BPRS-P	BPRS-Depression	CDSS	Clinician rated CAINS Experience	SAPS
**CAINS Expression Subscale**	[22]	0.13	0.01	0.15	-	-
**CAINS SR Expression Subscale**	[23]	0.14	-	0.31, p<0.05	0.34, p<0.01	-
**SANS Blunted Affect Subscale**	[29]	-	-	-0.12	-	-
**BPRS Anergia**	[21]	-	-	-	-	0.25 (SAPS Hallucinations); -0.04 (SAPS Delusions); 0.42, p<0.01 (SAPS Bizarre); 0.11 (SAPS Thought Disorder)

Divergent validity estimates of negative symptom subscales measuring blunted affect.

Included studies reporting on the convergent validity of the subscales measuring blunted affect is presented in Table 4. Studies that compared blunted affect subscales with similar subscales found that the BNSS Blunted Affect Subscale [19] as well as that of the SEB [33] have good convergent validity when compared with the SANS Blunted Affect Subscale (0.79 and 0.76 respectively). The BPRS Anergia Subscale (that measures blunted affect, emotional withdrawal, motor retardation and disorientation) correlated moderately with the SANS Blunted Affect Subscale (0.66). Similarly, the CAINS Expression correlated moderately with the SANS Blunted Affect Subscale (0.61). The CAINS-SR Expression subscale correlated poorly (0.29) with the Expression subscale of the original CAINS, again this not surprising given patients restricted awareness of their outward expression of emotion in the presence of others. Additionally, a few studies compared the subscales measuring blunted affect with overall negative symptom instruments. These studies found that the CAINS Expression Subscale correlated moderately with the BPRS-N (0.52, p<0.01) as well as with the SANS (0.55, p<0.05). The SANS Blunted Affect Subscale correlated poorly with the PANSS-N (0.45, p<0.01).

The divergent validity estimates of the subscales measuring blunted affect are presented in Table 5 below. The Expression Subscale of the clinician-rated and self-rated CAINS correlated minimally with the BPRS-P (respectively 0.13 and 0.14). The CAINS Expression Subscale and the SANS Blunted Affect Subscale correlated minimally with the CDSS (respectively 0.15 and 0.12). However the CAINS SR Expression Subscale, in comparison to the clinician-rated Expression Subscale, had a slightly stronger correlation (of 0.31) with the CDSS.

## Discussion

### Summary of findings

This systematic review we identified 10 instruments that measure blunted affect in schizophrenia:eight clinician-rated and two self-report instruments. All instruments measure blunted affect in addition to other negative symptoms.

### Administration of instruments

We found that none of the instruments are particularly time consuming to administer. Brief instruments are likely to be better accepted by patients, and less likely to be influenced by patients becoming easily distracted or fatigued.

### Instrument items

Instrument items determine how symptoms are defined [1] and in this review we found that blunted affect items common across all instruments measure are: gestures, facial expressions and vocal expressions. In addition to these items, the SANS includes *poor eye contact and inappropriate affect*. It is suggested that the items of *poor eye contact* [29] and *inappropriate affect* [33] do not measure blunted affect, but more research is needed in this regard [29]. Cognisance should be taken of the fact that many of the subscales measuring blunted affect also include items measuring alogia as these two negative symptom clusters are grouped together as part of patients outward emotional expression [10].

### Psychometric properties

The overall negative symptom instruments as well as the subscales measuring blunted affect seem to be reliable. However, in terms of reliability we would like to highlight the following. Blunted affect, as is the case with other negative symptoms, may improve to some extent when acutely psychotic patients are on antipsychotic drugs. However, they frequently persist and display temporal stability in the post-acute phase of the illness. Studies included did not always specify the phase of illness so were unable to explore this further in our analysis of instruments’ test-retest reliability. Furthermore, *we are unable to answer why the overall CAINS has a lower internal consistency than the BNSS*. *As mentioned previously this difference cannot be ascribed to to scale length or differences in sample characteristics*. The poor internal consistency of the CAINS Self-report Expression Subscale is not surprising given the nature of blunted affect. Patients tend to be unaware of their restricted emotional expression [22]. Blunted affect, as is the case with other negative symptoms, may improve to some extent when acutely psychotic patients are on antipsychotic drugs.

Regarding the validity of the instruments, those assessing negative symptoms have moderate to good convergent validity and do not measure positive symptoms or depression (divergent validity). Some of the blunted affect subscales have good convergent validity when compared with similar subscales. For example, the BNSS Blunted Affect Subscale as well as RSEB Blunted Affect Subscale [33] correlated well with the SANS Blunted Affect Subscale. The BPRS Anergia Subscale has moderate convergent validity with the SANS Blunted Affect Subscale, but should be interpreted with caution, since the former subscale does not exclusively measure blunted affect. It is therefore not possible to determine how the blunted affect items of the Anergia Subscale in particular compare to those of the SANS. A similar issue arises when interpreting the moderate convergent validity of the clinician-rated and self- report CAINS Expression with the overall BPRS-N and SANS. Again the subscales were compared with overall negative symptom instruments making it difficult to interpret which blunted affect items converged with items in the overall negative symptom instruments.

The poor convergent validity between the clinician-rated and self-reported CAINS Expression Subscale is to be expected given the discrepancy between patients’ expressive emotions and their internal emotional experience [35]. In other words, patients may not perceive their expressive behaviours as blunted. Our findings suggest that self-report instruments do not provide an accurate picture of patients’ expressive behaviour. This supports the opinion of the developers of the CAINS Self-report who argue that the expression subscale will most probably be left out of newer versions of the instrument [23]. However, it should be noted that our systematic review is limited in that we only included two self-report instruments. We were unable to include the Subjective Experience of Negative Symptoms (as with the PNS-Q this instrument is based on the SANS) developed by Selten et al. [36], since the instrument’s validity was not reported on.

## Conclusion

All the clinician-rated instruments included in this review had good reliability and validity. The two self-report instruments are not effective in measuring patients’ understanding and awareness and self-perception of blunted affect. However, at this point in time there is too much uncertainty about the nature of blunted affect to conclude that patients’ understanding of blunted affect should not be measured. Future research into the awareness of blunted affect symptoms in patients with schizophrenia may help us to make more informed decisions about the role of self-report instruments. In addition to clinician-rated and self-report instruments, we foresee that increasingly more objective blunted affect instruments will be used in the future. For example, researchers [37–39] from the University of Pennsylvania (in the United States) have done extensive work on computer-based instruments that are able to assess the subtle facial expressions associated with blunted affect and quantify these expression in schizophrenia patients [37].

## Supporting Information

S1 PRISMA Checklist A checklist of PRISMA guidelines. (DOC)

S1 Table Blunted affect Items. Items assessing blunted affect in negative symptom instruments. (DOCX)
