# Supplementary material for: Instruments Measuring Blunted Affect in Schizophrenia: A Systematic Review
Source: PLoS One. 2015 Jun 2;10(6):e0127740. doi: 10.1371/journal.pone.0127740 (PMC4452733; doi:10.1371/journal.pone.0127740)
Supplement: S1 Table — (DOCX) [file pone.0127740.s002.docx]

**Supporting information**

**S1.Table. Blunted affect items.**

| **Instrument** | **No of Blunted Affect Items** | **Blunted Affect Items** |
| --- | --- | --- |
| **BNSS [1]** | 3 | Facial expression, Vocal expression, Expressive gestures. |
| **BPRS [2-3]** | 3 | Reduced emotional tone, Reduction in formal intensity of feelings, Flatness. |
| **CAINS [4]** | 3 | Facial Expression, Vocal Expression, Expressive Gestures. |
| **CAINS-SR [5]** | 5 | The instrument includes 5 emotional blunting items asking patients to talk about their verbal and non-verbal expressions. Nonverbal expression refers to facial expression and gestures, verbal expression refers to how talkative patients were. |
| **NSA-16 [6]** | 3 | Affect: reduced modulation, Affect: reduced display, Reduced expressive gestures. |
| **PANSS-N [7]** | 1 | In the PANSS-N, the emotional blunting item refers to diminished emotional responsiveness as observed through a reduction in facial expression, modulation of feelings, and communicative gestures. Observations are made of the physical manifestations of affective tone and emotional responsiveness during the course of interview. |
| **SANS [8]** | 8 | Unchanging facial expression, Decreased spontaneous movement, Paucity of expressive gestures, Poor eye contact, Non-responsiveness, Lack of vocal inflections, Inappropriate affect, Global rating of affective flattening. |
| **PNS-Q [9]** | 7 | People tell me that my tone of voice does not reflect my real feelings, I usually do not outwardly express my feelings, I have difficulty maintaining eye contact with others, When others laugh from jokes on TV, I usually do not, I cannot enjoy or be enthusiastic about things, I often do not know what I am feeling, People say that I look "frozen". |
| **MASS [10]** | NA | During a structured interview a participant’s hand co-verbal gestures and spontaneous smiles are recorded and counted. Specific rules applied when raters count hand co-verbal gestures. Post-interview participant has to smile and this posed smile is then rated. |
| **SEB [11]** | 16 | Affect: Absent, shallow, incongruous mood. Constricted affect (narrow range). Unvarying affect (lacks modulation). Unrelated affect (lacks warmth, empathy). Behaviour: Expressionless face. Unvarying, monotonous voice. Seclusive/withdrawn, avoids social contact. Lacks social graces (neglect dress, ill-mannered, unbathed). Difficult to excite emotions/unresponsive. Lacks spontaneity. Causeless, silly laughter/disposition. Indifferent to surroundings (staff, visitors…) Thought content: Indifference/lack of affection for family, friends. Indifference/unconcern for own present situation. Indifference/unconcern for own future (lacks plans, ambition, drive, desires). Paucity of thought (unable to elaborate on answers). |
| **SEB [12]** | 8 | Constricted affect; Unvarying affect; Expressionless face; Unvarying monotonous voice; Difficulty to excite emotions. Lacks spontaneity, Paucity of thought .Unrelated affect. |
| **SEB [13]** | 6 | Absent, shallow, incongruous mood; Constricted affect; .Unvarying affect; Expressionless face; Unvarying monotonous voice; and Difficulty to excite emotions. |

Items assessing blunted affect in negative symptom instruments.

**References**

1. Kirkpatrick B, Strauss GP, Nguyen B, Fischer A, Daniel DG, Cienfuegos A, et al. The Brief Negative Symptom Scale: Psychometric Properties. Schizophrenia Bulletin. 2010;37(2): 300–305.

2. Gur RE, Mozley D, Resnick SM, Levick S, Erwin R, Saykin AJ, et al. Relations among clinical scales in schizophrenia. The American Journal of Psychiatry. 1991;148 (4):,472-478.

3. Overall JE, Gorham DR (1962) The Brief Psychiatric Rating Scale. Psychological Reports: 10, 799-812.

4. Kring AM, Gur RE, Blanchard JJ, Horan WP, & Reise SP. The clinical assessment interview for negative symptoms (CAINS): Final development and validation. American Journal of Psychiatry. 2013;170*:*165-172.

5. Park SG, Llerena K, McCarthy JM, Couture SM, Bennettt ME, & Blanchard JJ. Screening for negative symptoms: Preliminary results from the self-report version of the Clinical Assessment Interview for Negative Symptoms. Schizophrenia Research. 2012;135:139-143.

6. Alexrod B, Goldman RS, & Alphs LD. Validation of the16-item negative symptom assessment. J Psychiat Res. 1993;27(3)*:* 253-258.

7. Kay SR, Fizbein A, Opler LA. The Positive and Negative Syndrome Scale (PANSS) for Schizophrenia. Schizophr Bull. 1987;13: 261-267.

8. Rabany L, Weiser M, Werbeloff N, & Levkovitz Y. Assessment of negative symptoms and depression in schizophrenia: Revision of the SANS and how it relates to the PANSS and CDSS. Schizophrenia Research. 2011;126:226-230.

9. Iancu I, Poreh A, Lehman B, Shamir E, & Kotler M. The positive and negative symptom questionnaire: a self-report scale in schizophrenia. Comprehensive Psychiatry. 2005;46:61-66.

10. Tremeau F, Goggin M, Antonius D, Czobor P, Hill V, & Citrome L. A new rating scale for negative symptoms. The Motor-Affective-Social Scale. Psychiatry Research. 2008;160: 346-355.

11. Abrams R, & & Taylor MA. A rating scale for emotional blunting. American Journal of Psychiatry. 1978;:135(2)*:* 226-9.

12. De Leon J, Peralta V, & Cuesta MJ. Negative symptoms and emotional blunting in schizophrenic patients. Journal of Clinical Psychiatry. 1993;54(3):103-108.

13. Berenbaum SA, Abrams R, Rosenberg S, & Taylor MA . The nature of emotional blunting: A factor-analytic study. Psychiatry Research. 1985;20: 57-67.
